# Human miR-221/222 in Physiological and Atherosclerotic Vascular Remodeling

**DOI:** 10.1155/2015/354517

**Published:** 2015-06-29

**Authors:** Dmitry A. Chistiakov, Igor A. Sobenin, Alexander N. Orekhov, Yuri V. Bobryshev

**Affiliations:** ^1^Department of Medical Nanobiotechnology, Pirogov Russian State Medical University, Moscow 117997, Russia; ^2^The Mount Sinai Community Clinical Oncology Program, Mount Sinai Comprehensive Cancer Center, Mount Sinai Medical Center, Miami Beach, FL 33140, USA; ^3^Laboratory of Angiopathology, Institute of General Pathology and Pathophysiology, Russian Academy of Sciences, Moscow 125315, Russia; ^4^Laboratory of Medical Genetics, Russian Cardiology Research and Production Complex, Moscow 121552, Russia; ^5^Institute for Atherosclerosis Research, Skolkovo Innovative Center, Moscow 121609, Russia; ^6^Faculty of Medicine and St Vincent's Centre for Applied Medical Research, University of New South Wales, Sydney, NSW 2052, Australia; ^7^School of Medicine, University of Western Sydney, Campbelltown, NSW 2560, Australia

## Abstract

A cluster of miR-221/222 is a key player in vascular biology through exhibiting its effects on vascular smooth muscle cells (VSMCs) and endothelial cells (ECs). These miRNAs contribute to vascular remodeling, an adaptive process involving phenotypic and behavioral changes in vascular cells in response to vascular injury. In proliferative vascular diseases such as atherosclerosis, pathological vascular remodeling plays a prominent role. The miR-221/222 cluster controls development and differentiation of ECs but inhibits their proangiogenic activation, proliferation, and migration. miR-221/222 are primarily implicated in maintaining endothelial integrity and supporting quiescent EC phenotype. Vascular expression of miR-221/222 is upregulated in initial atherogenic stages causing inhibition of angiogenic recruitment of ECs and increasing endothelial dysfunction and EC apoptosis. In contrast, these miRNAs stimulate VSMCs and switching from the VSMC “contractile” phenotype to the “synthetic” phenotype associated with induction of proliferation and motility. In atherosclerotic vessels, miR-221/222 drive neointima formation. Both miRNAs contribute to atherogenic calcification of VSMCs. In advanced plaques, chronic inflammation downregulates miR-221/222 expression in ECs that in turn could activate intralesion neoangiogenesis. In addition, both miRNAs could contribute to cardiovascular pathology through their effects on fat and glucose metabolism in nonvascular tissues such as adipose tissue, liver, and skeletal muscles.

## 1. Introduction

In the body, the vascular system fulfills a variety of vital functions. Blood vessels transport nutrients and oxygen to every cell and take off wastes and carbon dioxide. The vasculature is involved in maintaining body temperature, pH, and mineral homeostasis. Blood and lymph vessels transfer immune cells essential for host defense against various pathogens. The vascular network is composed of differently sized vessels like the micro-, small, medium, and large vessels.

The vascular tissues include several types of cells including endothelial cells (ECs), vascular smooth muscle cells (VSMCs), pericytes, fibroblasts, resident macrophages, resident mesenchymal stem cells (MSCs) and progenitors, and connective tissues. The classical three-layer structure of the vascular wall involves the intima, media, and adventitia flanked with the elastic laminae [1].

The vessel wall is adopted for various functional requirements that could be changed depending on each segment of the circulatory system. For example, aorta and large arteries are enriched with elastic fibers in order to support sufficient flow and pressure for delivery of blood components to the extreme peripheral tissues [2]. The circulatory network is a dynamic system that constitutively develops and matures to be better adapted to the rapid changes in microenvironmental conditions. Physiological processes associated with structural modifications in the vascular wall and related to vascular developmental changes during embryogenesis and adaptive responses such as neovascularization are termed vascular remodeling [3, 4].

In embryogenesis, vascular remodeling is an essential mechanism that supports the development and formation of the mature vascular network. In humans, vascular remodeling starts at day 21 when the immature heart begins to beat pushing blood through the early vasculature [5]. Indeed, biomechanical and hemodynamic forces and characteristics such as shear stress, cylinder stress, pressure, velocity, and flow become applicable to the developing vessels and induce signaling cascades that contribute to angiogenesis, vessel sprouting, vascular branching, hierarchy, maturation, and arterial-vein identity [6]. These signaling pathways are activated in ECs and VSMCs through the mechanism of mechanotransduction associated with upregulation and downregulation of certain genes involved in vasculogenesis, cell differentiation, proliferation, migration, differentiation, adhesion, and cell-matrix interactions [7].

To date, mouse is a leading model system for studying the physical and molecular regulation of vascular development and embryogenic vascular remodeling. In cultured mouse ECs, mechanical shear stress was shown to activate a signal transduction complex composed of three receptors such as vascular endothelial growth factor receptor 2 (VEGFR2)/fetal liver kinase 1 (FLK1), vascular endothelial cell cadherin (VE-cadherin), and platelet endothelial cell adhesion molecule 1 (PECAM1). As a result, cells realigned parallel to the direction of flow become mechanosensitive [8]. Direct transmission of mechanical force occurs through PECAM1 to VE-cadherin that acts as an adaptor protein activating Flk1 and catalyzing the activation of the phosphatidylinositol-3-OH kinase (PI3K) signaling cascade [9]. This leads to the induction of a panel of transcription factors including Krüppel-like factors- (KLF-) 4 and 6 and T cell acute lymphocytic leukemia 1 (Tal1) essential for both hematopoiesis and vasculogenesis [10, 11].

Vascular remodeling is a crucial mechanism of the routine endothelial replacement and repair of damaged vessel wall in order to maintain vascular integrity and function and prevent thrombosis [12]. This process involves many cell types including resident and nonresident stem and nonstem cells. In arterial injury, bone mesenchymal stem cells were shown to have a capacity to differentiate to neo-ECs and contribute to reendothelization [13]. The reendothelization is controlled by bone-marrow derived transcription factor KLF-10 [14]. Medial VSMCs and adventitial fibroblasts undergo phenotypic changes associated with induction of proliferation, migration, and differentiation and enhanced production of extracellular matrix proteins and adhesion molecules and release of reactive oxygen species, chemokines, cytokines, growth factors, and matrix metalloproteinases (MMPs) that, collectively, affect medial VSMC contractility and growth directly and that stimulate recruitment and retention of circulating inflammatory and progenitor cells to the vascular wall [15]. Pericytes characterized with significant phenotypic plasticity and ability to transdifferentiate to several vascular cell types such as VSMCs, fibroblasts, and macrophages also contribute to vascular repair [16].

However, vascular remodeling can occur as a maladaptive response against vessel injury. In that case, the remodeling could be associated with various vascular abnormalities such as endothelial dysfunction, hypertrophy, or fibrosis and be a part of a pathophysiological mechanism in the development of hypertension, thrombosis, restenosis, cardiomyopathy, atherosclerosis, and other cardiovascular disease.

Atherosclerosis is characterized with marked structural alterations in the arterial wall induced by the subendothelial accumulation of modified lipoproteins followed by the chronic inflammatory response [17, 18]. Vascular remodeling results from a close interplay of changes in the vascular tone and structure. An important concept for arterial remodeling (so-called Glagov's phenomenon) considers that arteries remodel to maintain constant flow despite increases in atherosclerotic lesion mass [19]. Indeed, atherosclerotic vascular remodeling is an adaptive response of the circulatory system to the growth of atherosclerotic plaques [3].

Atherogenic vascular remodeling involves various cell types, with a prominent role of ECs and VSMCs. Inflammatory monocytes and macrophages recruited in the atherosclerotic plaque contribute to the atherosclerosis-associated changes in the vascular wall via stimulation of ECs and VSMCs [20]. Briefly, inflammatory stimuli and changes in hemodynamic characteristics of blood flow in atherosclerotic vessels such as elevated blood pressure and increased shear stress led to the proinflammatory activation of arterial endothelium and advanced apoptosis of ECs, with subsequent denudation of the arterial wall and increased risk of thrombosis [21, 22]. In contrast, VSMCs lack their quiescence and “contractile” phenotype and acquire capacity to proliferate and migrate. This leads to the neointimal formation associated with intimal thickening and ectopic calcification of atherosclerotic arteries [23]. Vascular remodeling is accompanied with upregulation or downregulation of various molecular subsets including microRNAs (miRNAs).

Within the last decade, a research focused on evaluation of a role of miRNAs in physiological and pathological structural changes in vessels becoming more and more intensive [24]. This especially concerns studying involvement of tissue-specific (e.g., vascular or heart) miRNAs that normally control embryonic angiogenesis, cardiogenesis, and/or vascular repair. Human miRNA- (miR-) 221 and its paralogue miR-222 show notable activities in the vascular network by influencing angiogenic properties of ECs [25] and phenotypic changes in VSMCs [26]. miR-221/222 have been found to regulate essential physiological vascular processes such as angiogenesis [27], neointimal hyperplasia [28], vessel wound healing [29], and vascular aging [30]. Furthermore, this miRNA is involved in a variety of vascular-related pathological mechanisms including tumor angiogenesis [31], atherosclerotic inflammation and vascular remodeling [32], fibrosis [33], vascular calcification [34], cardiac hypertrophy [35], angiotensin II dependent hypertension [36], and diabetic hyperglycemia-induced endothelial dysfunction [37]. In this review, we provide the information about the contribution of miR-221/222 to vascular remodeling in normal and atherosclerotic vessels.

## 2. Biogenesis and mRNA-Silencing Function of miR-221/222

**Figure 1:**
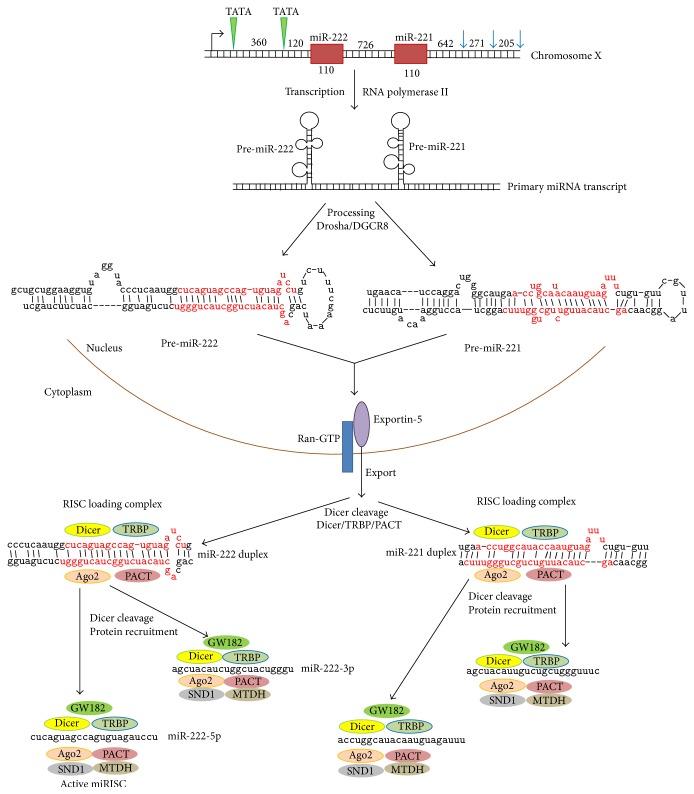
Biogenesis of human miR-221/222. miRNA-encoding genes are shown in red boxes. In the common promoter, TATA boxes are marked by green triangles. Poly(A) signals are displayed with blue arrows. Numbers designate the distance between the regulatory transcription elements (in bp) and the length of each miRNA gene and intergenic spacer. The sequence of a mature miRNA duplex is presented in red color. Ago2: Argonaute-2; DGCR8: DiGeorge syndrome critical region gene 8; GW182: glycine-tryptophan protein of 182 kDa; MTDH: metadherin; PACT: protein kinase R-activating protein; Ran-GTP: GTP-binding nuclear protein Ran; SND1: staphylococcal nuclease domain-containing protein 1; TRBP: TAR RNA-binding protein.

In human DNA, the miR-221/222 gene cluster is located on chromosome Xp11.3 [38]. The miR-221 and miR-222 genes are separated by a distance of 726 bp (Figure 1). Nucleotide sequences of both genes share high similarity to each other. In fact, the genes are paralogues arisen from the duplication of the ancestral gene. They are transcribed as a single long noncoding RNA precursor with RNA polymerase II [39]. The promoter region contains two canonical TATA boxes located on 550 and 190 base pairs (bp) upstream of pre-miR-222. Three poly(A) signals are located downstream of pre-miR-221. The expression of the miR-221/222 gene cluster is positively regulated by angiotensin II [36] and downregulated by a repressive complex formed by estrogen receptor *α* and two nuclear receptor corepressors NCOR1 and NCOR2 [38].

In the nucleus, the common pri-miR-221/222 transcript then is spliced and split by the “microprocessor” complex Drosha/DiGeorge syndrome critical region gene 8 (DGSR8) [40] with formation of the individual pre-miR-221 and pre-miR-222 precursors that both have the length of 110 nucleotides (nt). Pre-miRNAs are transferred to the cytoplasm by the nuclear transporter exportin-5, a Ran-GTP RNA-binding protein [41], where Dicer cleaves the miRNA precursors to mature miRNA duplexes. Each miRNA duplex is stabilized with the heterotrimeric complex Argonaute-2 (Ago2)/TAR RNA-binding protein (TRBP)/protein kinase R-activating protein (PACT) [42] that is a core of the RNA-induced silencing complex (RISC). Double stranded miRNA is generally a transient imperfect duplex molecule consisting of a passenger strand and a mature miRNA strand (also referred to as guide strand) [43]. The duplex was then cleaved by Dicer with the formation of a functional single stranded mature miRNA bound to the RISC complex (miRISC). During miRISC complex formation, several additional proteins are recruited including GW182 (glycine-tryptophan protein of 182 kDa), MTDH (metadherin), and SND1 (staphylococcal nuclease domain-containing protein 1). GW182 contains two repression domains, which trigger translational repression with mild effect on mRNA decay, and multiple glycine/tryptophan repeats essential for formation and maintaining stability of the multiprotein RISC complex [44]. SND1 is a coactivator of several eukaryotic transcription factors and a component of ribonucleoprotein complexes such as spliceosome and RISC complex that increases their functional efficiency and kinetics [45]. In the RISC complex, MTDH acts as a scaffold protein possessing RNA-binding properties [46]. Generally, only one strand is incorporated into the miRISC selected on the basis of its thermodynamic instability [47]. Indeed, maturation of the pre-miR-221 results in the formation of either mature 23nt-long miR-221-5p or miR-221-3p. For miR-222, generating two mature 21nt-long miRNAs (miR-222-5p and miR-222-3p) is possible (Figure 1).

Following assembly of the miRISC complexes, there is increasing evidence that miRNAs exert their posttranscriptional regulatory functions in the context of processing bodies (P-bodies). P-bodies are distinct compartments (foci) in the cytoplasm involved in RNA degradation and turnover [48]. Furthermore, P-bodies were recognized to serve as the functional site of miRNA-mediated gene silencing since duplex miRNA-containing RISC complexes were found in these compartments [49]. The miRISC complex binds target mRNA through Watson-Crick base pairing between the guide strand and the 3′ untranslated region (UTR) of the target [50]. The precision of target recognition heavily depends on base pairing between the seeds (nt 2–8 at the 5′ end) of the miRNA guide [51].

Extensive base pairing between the miRNA guide and mRNA target induces the degradation of mRNA target through Ago2-mediated cleavage and deadenylation that is dependent on the activity of the complex comprised by Pop2 (pyrin domain containing 2), Ccr4 (mRNA deadenylase), and Not1 (CCR-NOT transcription complex subunit 1) [52]. Deadenylated mRNA is then degraded with exoribonuclease Xrn1p and decapped with mRNA-decapping (Dcp) enzymes [53]. In the cytoplasm, mRNAs actively translated by polyribosomes are targeted by miRISC complexes to suppress translation [54]. These mRNAs could then be delivered to P-bodies for degradation or storage in order to preserve a pool of transcripts whose translation is rate-limited for cell growth [55].

## 3. miR-221/222 Function in Physiological Vascular Remodeling

### 3.1. Antiangiogenic Activity of the miR-221/222 Cluster in Vascular ECs

Inside the vascular tissue, ECs are responsible for critical functions [56, 57]. They provide inner lining to the blood vessels and the heart, secrete a variety of bioactive molecules to affect the local environment of the cells, and transfer the molecules from the blood to the interstitial fluid. During the developmental stages and in diseases, ECs tightly cooperate with the nearby muscles in mediating angiogenesis and neovascularization. Overall, ECs contribute to the maintenance and growth of normal tissues [56, 57].

It was shown that human blood cord-derived CD34+ HPCs, endothelial progenitor cells (EPCs) [58], and human umbilical vein endothelial cells (HUVECs) [59] as well as quiescent ECs [60] highly express miR-221/222 thereby suggesting a critical role of this miRNA cluster in regulating development and function of the vascular endothelium. However, in vascular ECs, the role of miR-221/222 greatly varies depending on the developmental stages and microenvironment.

ECs were shown to express both SCF and its receptor c-kit [60–63]. Indeed c-kit-dependent angiogenic properties of ECs are targeted by miR-221/222 [25]. miR-221/222 were shown to be transcribed in c-kit-positive HUVECs suggesting a coordinated transcriptional regulation. miR221/222 downregulates c-kit on the posttranslational level since reduction in c-kit protein levels but not in mRNA levels was observed in HUVECs transfected with miR221/222 [25].

In embryonic ECs, miR-221 was shown to drive angiogenesis and especially proliferation and migration of tip ECs in sprouting neovessels [64]. In miR-221-deficient zebrafish embryos, proliferation of ECs is blocked [65]. miR-221 supports embryonic angiogenesis through targeting two key regulatory molecules, CDKN1B and PI3KR1. CDKN1B is a cyclin-dependent kinase (CDK) inhibitor p27^Kip1^ that negatively regulates cell growth and prevents proliferation by inhibiting cyclin-dependent kinases [66]. PI3KR1 is the p85-regulatory subunit *α* of the phosphoinositide-3-kinase (PI3K) complex, an essential component of PI3K signaling that is crucial for vascular development [67]. PI3KR1 inhibits the PI3K p110 catalytic subunits but is also necessary for activity of the membrane-bound enzyme and stimulation by a receptor tyrosine kinase [68]. miR221 is likely to alter an appropriate balance in regulatory and catalytic subunits of PI3K that may indeed affect the receptor tyrosine kinase-mediated activation of PI3K and local PI3K activity in separate subcellular compartments such as growing filopodia of tip embryonic ECs.

miR-221 directs proliferation of embryonic ECs through vascular endothelial growth factor C (VEGF-C)/Fms-related tyrosine kinase 4 (Flt4) signaling [64] that is modulated by PI3K3R1 that stimulates interaction of PI3K with VEGF-C-activated Flt4 (VEGFR3), a receptor for VEGF-C and VEGF-D [69]. Activation of notch signaling in stalk ECs was shown to inhibit both Flt4 and miR-221 that leads to increase in CDKN1B levels and reducing proliferation [64]. Specification of hemogenic ECs from the primordial endothelium is controlled by c-kit, notch signaling, and p27^Kip1^-dependent cell cycle control [66]. miR-221 is involved in crosstalk with all signaling components essential phenotypic definition of hemogenic ECs and therefore plays a key role at early differentiation stages of the vascular endothelial development.

**Figure 2:**
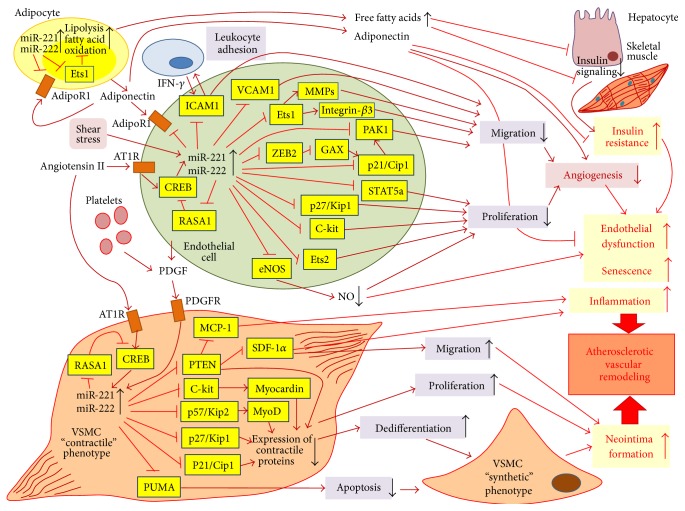
Effects of miR-221/222 on vascular endothelium and vascular smooth muscle cells (VSMCs) in atherosclerotic vascular remodeling. In arterial endothelial cells (ECs), expression of miR-221/222 could be upregulated by angiotensin II and shear stress. miR-221/222 are able to positively regulate expression through suppression of RAS p21 protein activator 1 (RASA1), an inhibitor of CREB (cAMP response element-binding protein) that drives angiotensin II induced expression of both miRNAs. Increased levels of miR-221/222 suppress angiogenic activation of quiescent terminally differentiated ECs through inhibiting endothelial proliferation and migration. Proliferation is suppressed via negative regulatory effects of the miR-221/222 cluster on several key genes such as cyclin-dependent kinase cell cycle regulators p21^Cip1^ and p27^Kip1^, transcription factors Ets1 and Ets2, signal transducer and activator STAT5a, and receptor for mast/stem cell growth factor c-kit. Notably, miR-221/222 downregulate expression of endothelial NO-synthase (eNOS) that lead to lowered production of nitric oxide (NO), an important modulator of function and proliferation of vascular ECs. Decreased NO production contributes to endothelial dysfunction and promotes EC senescence. miR-221/222 could downregulate p21^Cip1^ either directly or through blocking of ZEB2 (zinc finger E-box binding homeobox 2), which represses translation of mesenchyme homeobox 2 (MEOX2 or GAX), a transcriptional activator of p21^Cip1^. The miR-221/222 cluster attenuates EC migration by suppressing endothelial production of matrix metalloproteinases (MMPs) and several key adhesion modulators such as intercellular adhesion molecule-1 (ICAM-1), vascular cell adhesion molecule-1 (VCAM-1), integrin-*β*3, and serine/threonine-protein kinase PAK-1. The miR-221/222 cluster could probably diminish endothelial expression of adiponectin receptor AdipoR1. Adiponectin is produced by adipocytes and plays protective role for ECs by preventing endothelial dysfunction. In obesity, miR-221/222 are upregulated in the adipose tissue causing activation of lipid catabolism (lipolysis and fatty cell oxidation) through inhibition of Ets1, a transcription factor that controls expression of fatty acid synthase and other lipid-synthesizing enzymes. As a result, adipocytes release increased amounts of free fatty acids to blood that inhibit insulin signaling in liver and skeletal muscle inducing peripheral insulin resistance. Insulin resistance contributes to endothelial dysfunction. In VSMCs, expression of miR-221/222 is stimulated by angiotensin II and platelet-derived growth factor (PDGF) that is secreted by activated platelets and ECs in response to vascular injury. Upregulation of these miRNAs supports proliferation and increase mobility of VSMCs. In VSMCs, miR-221/222 inhibit several regulatory factors such as those of p21^Cip1^, p27^Kip1^, p57^Kip2^, c-kit, and phosphatase and tensin homolog (PTEN) that are crucial for differentiation and establishment of the contractile phenotype of VSMCs. p57^Kip2^ and c-kit activate MyoD and myocardin, two key transcription factors involved in myogenesis. Indeed, miR-221/222-dependent downregulation of expression of SMC-specific contractile proteins causes VSMC dedifferentiation and switch from the “contractile” to “synthetic” phenotype. By suppressing PTEN, miR-221/222 induce expression of several proinflammatory chemokines such as monocyte chemotactic protein-1 (MCP-1) and stromal cell-derived factor 1*α* (SDF-1*α*) that attract proinflammatory lymphocytes, dendritic cells, and macrophages to the inflamed site, for example, to the atherosclerotic plaque. In addition, miR-221/222 downregulate PUMA (p53 upregulated modulator of apoptosis), a critical apoptotic inducer thereby preventing apoptosis of VSMCs. Finally, dedifferentiated VSMCs become involved in neointima formation, an essential stage in atherosclerosis-associated remodeling of the arterial wall.

In EPCs, miR-221 was found to suppress serine/threonine-protein kinase PAK1 [70] (Figure 2). PAK1 belongs to the family of p21^Waf1/Cip1^-activated kinases and serves as a target for small GTPases Cdc42 and Rac [71]. EPCs are known to play an essential role in vascular repair and maintenance of vascular homeostasis through reendothelialization and neovascularization. EPCs transfected with miR-221/miR-222 loose capacity to do wound healing and tube formation [63].

PAK1 is involved in cytoskeleton reorganization and activation of EPC motility and proliferation is response to vascular injury [72, 73]. PAK1 was found to activate c-Raf/MEK/ERK signaling by phosphorylation of MEK on serine 298 [74] and RAF on serine 338 [75]. Therefore, miR-221-mediated PAK1 targeting attenuates proliferation of EPCs and impairs their function through downregulating c-Raf/MEK/ERK pathway thereby displaying antiangiogenic properties and contributing to maintenance of quiescent phenotype [70]. Interestingly, biologically active molecular components of garlic extract such as diallyl disulfide and diallyl trisulfide inhibit miR-221 expression and restore angiogenic properties of EPCs via derepressing c-kit and activating signaling protein kinases Akt and ERK 1/2 [58].

In mature human ECs, miR221/222 exhibit strictly antiangiogenic properties mediated by many targets. This mRNA cluster could display the antiangiogenic effects through inhibiting c-kit, transcription factors Ets1, Ets2 [25], zinc finger E-box binding homeobox 2 (ZEB2) [76], signal activator and transducer 5A (STAT5a) [60], and endothelial NO-synthase (eNOS) [77, 78]. Interestingly, Kaposi sarcoma-associated herpes virus (KSHV) oncogenes such as latent nuclear antigen (LANA) and kaposin B were shown to activate EC motility and proliferation essential for tumor neoangiogenesis by derepressing Ets1 and Ets2 [79, 80] through direct transcriptional suppression of miR-221/222. Ets1, a prototype of the Ets family of transcription factors, supports EC-mediated angiogenesis by inducing expression of MMPs and integrin-*β*3 in ECs [81] (Figure 2). eNOS, which plays a crucial role in the regulation of vascular function, contributes to angiogenesis by production of nitric oxide, a vasoactive molecule, in response to multiple stimuli [82].

In epithelial tissues, ZEB2 known as a regulator of epithelial-to-mesenchymal transition (EMT) acts as a stimulator of cell proliferation and mobility [83]. ZEB2 mRNA is directly targeted by miR-221 at the consensus site located at the 3′UTR [76]. On the other hand, ZEB2 acts as a translational repressor of mesenchyme homeobox 2 (MEOX2 or GAX), a transcriptional factor that is expressed in quiescent vascular ECs and inhibits EC transition to the angiogenic phenotype in response to proangiogenic growth factors [84]. In ECs, MEOX2 activates expression of p21^WAF1/CIP1^ by binding directly to its promoter and enhancer [85] that causes cell cycle arrest in G0/G1. MEOX2 is also able to suppress EC activation through the downregulation of activity of the nuclear factor NF-*κ*B [86].

STAT5A was shown to mediate angiogenic activation of ECs through several mechanisms including Src/Jak2-dependent stimulatory signals from fibroblast growth factors FGF2 and FGF8b [87]. Prolactin family members such as proliferin and prolactin could mediate proangiogenic properties of the FGF/STAT5A activating axis through autocrine mechanism [88]. FGF2-mediated activation leads to the recruitment of STAT1 and STAT5A and to a lesser content STAT3 in ECs followed by prolactin-induced production of VEGF, a potent proangiogenic factor able to promote angiogenesis via the positive autocrine feedback loop [89]. Interleukin- (IL-) 3 induces endothelial mitogenic proliferation by binding to the IL-3 receptor and recruitment of STAT5A and STAT5B [90]. IL-3-mediated activation of vascular ECs is accompanied with a secondary release of platelet-activating factor (PAF) that stimulates EC motility but not proliferation.

ECs were found to spontaneously respond to the vascular injury by induction of the proangiogenic transcriptional program and changes in the cellular phenotype and behavior. For example, exposure of human hepatic sinusoidal ECs to clinical doses of ionizing radiation results in upregulation of AMP-activated protein kinase (AMPK) and p38 mitogen-activated protein kinase- (MAPK-) mediated production of MMP-2 and VEGFR2 needed to degrade the extracellular matrix and basement membrane and activate ECs towards tube formation [91]. Irradiated HUVECs were observed to induce expression of angiogenesis-related miRNAs including the miR-221/222 cluster [92] that does not support neoangiogenesis itself but contributes to stabilization and integrity of already assembled neovessels. Indeed, in ECs, the miR-221/222 cluster shows antiangiogenic activity and prevents endothelial activation towards vascular remodeling and neovascularization. miR-221/222 are responsible for establishing quiescent phenotype of ECs and maintaining homeostasis of the vascular endothelium.

In consistence with this, senescent human aortic ECs were shown to express higher levels of antiangiogenic miR-221 and miR-222 [93] associated with reduced synthesis and activity of eNOS and increased production of caveolin-1, a negative eNOS regulator [30]. However, rhesus macaque mesenchymal stem cells (MSCs) derived from the bone marrow of aged animals displayed decreased expression of miRNA-221 compared with young monkeys [94]. In older humans, miR-221 expression in peripheral blood mononuclear cells (PMBCs) was also reduced while expression of its targets c-kit and PI3KR1 was increased with age [95]. Indeed, miR-221/222 could contribute to replicative senescence and cell aging but their lifespan-associated effects seem to be cell specific. Upregulation of miR221/222 in senescent ECs could be a consequence of age-related endothelial dysfunction and stimulatory effects of low-grade inflammation commonly persisted in the wall of aged vessels.

### 3.2. miR-221/222-Mediated Regulation of Phenotypic Plasticity of VSMCs

VSMCs are the prevalent cell type in the arterial tunica media. They are primarily involved in the regulation of vascular tone. A notable feature of VSMCs is their significant phenotypic plasticity and ability to differentiate/dedifferentiate in response to various physiological stimuli and pathological stresses such as vessel wounding or vascular pathology [29]. Quiescent VSMCs rarely proliferate and have a contractile phenotype characterized by expression of SMC-specific genes including smooth muscle *α*-actin (*α*-SMA), transgelin (TAGLN or SM22*α*), smooth muscle myosin heavy chain (SM-MHC), *α*- and *β*-tropomyosins, *α*1-integrin, caldesmon, and calponin [96]. During dedifferentiation, VSMCs downregulate expression of contractile markers and acquire a synthetic phenotype associated with enhanced synthesis of collagens and MMPs along with induction of proliferation and motility [97]. VSMC-mediated neointimal hyperplasia plays a central and universal role in response to vascular injury and wound healing. In neointimal hyperplasia, VSMCs actively proliferate and migrate from the tunica media primarily to the tunica intima causing arterial wall thickening [98]. However, an aberrant proliferation of VSMCs is frequently involved in the pathogenesis of vascular proliferative diseases such as atherosclerosis, postangioplasty or in-stent restenosis, and transplant vasculopathy [99]. In atherosclerotic intima, the structural and functional changes of VSMCs lead to the formation of so-called secretory phenotype of VSMCs [100, 101].

miR-221/222 are highly expressed in VSMCs [32, 102]. Knockdown of miR-221 and miR-222 reduces VSMC proliferation* in vitro* and inhibits neointimal hyperplasia-induced intimal thickening in rat carotid artery after vascular injury [103]. Interestingly, the miR-221/222 cluster exhibits opposite activities towards vascular ECs and VSMCs. In ECs, these miRNAs inhibit proliferation and migration and cause proapoptosis. In VSMCs, both miRNAs stimulate proliferation and cell mobility and induce antiapoptosis [102]. miR-221/222 play a key role in triggering VSMC dedifferentiation and switching from the contractile phenotype to synthetic phenotype [102]. The miR-221/222 cluster was reported to be markedly upregulated in quiescent VSMCs in response to vascular injury and in proliferative VSMCs [102]. In VSMCs, the miRNA cluster expression in response to injury is induced by platelet-derived growth factor (PDGF) [26].

PDGF is released from platelets and endothelial cells at sites of vascular injury [104]. In VSMCs, PDGF-induced miR-221/222 expression leads to the inhibition of several cell cycle regulators such as p27^Kip1^, p57^Kip2^, and c-kit [26, 102]. miR-221-dependent downregulation of p27^Kip1^ is crucial for PDGF-mediated induction of cell proliferation. Interestingly, miR-221 and Skp2 (S-phase kinase-associated protein 2) were shown to temporally regulate p27^Kip1^ in VSMCs: miR-221 in G1-phase while Skp2 in S-phase associated with cell growth [105]. Skp2 is a component of the ubiquitin protein ligase complex (SKP1-cullin-F-box) that is involved in the control of cell cycle progression via degradation of CDKs, cyclins, and CDK inhibitors including p27^Kip1^ [106].

Like p27^Kip1^, p57^Kip2^ (cyclin-dependent kinase inhibitor 1C, CDKN1C) suppresses several G1 cyclin/Cdk complexes preventing cell proliferation [107]. In addition, p57^Kip2^ stabilizes and activates MyoD, a myogenic regulatory transcription factor, essential for muscle differentiation [108]. miR-221-induced downregulation of p57^Kip2^ results in inactivation of MyoD and prevention of VSMC differentiation towards the contractile phenotype (Figure 2). Decreased c-Kit causes inhibition of SMC-specific contractile gene transcription program by reducing expression of Myocardin (Myocd), a potent SMC-specific nuclear coactivator [109]. Indeed, miR-221 mediates PDGF-induced phenotypic changes in VSMCs by blocking expression of SMC contractile genes and stimulating VSMC dedifferentiation, proliferation, and migration.

## 4. Role of miR-221/222 in Atherosclerotic Vascular Remodeling

### 4.1. Inhibitory Effects of miR-221/222 on ECs in Atherosclerosis-Associated Vascular Remodeling

In mammals, the cardiovascular function is greatly influenced by the interaction between the hemodynamics and the vascular endothelium. Shear stress is created by blood flow when by blood flow affects the endothelium. Shear-induced mechanotransduction (e.g., conversion of mechanical stresses to biochemical responses) is especially influential in elastic vessels such as aorta and arteries where blood flow controls vascular tone and structure [110]. Mechanical stimulation of vascular endothelium with shear stress results in release of endothelial-derived vasoactive substances such as NO, growth factors, prostaglandins, and so forth. Sustained changes in local hemodynamics stimulate adaptive structural remodeling of the artery wall through coordinated changes in expression of multiple vascular proteins.

Shear stress induces phenotypic changes in the vascular endothelium in specific aortic and arterial sites that in turn specify whether this site will be resistant or prone to proatherogenic changes and progression to the atherosclerotic plaque [111, 112]. In principal, intensive shear stress is beneficial since it promotes adaptive dilatation or structural arterial remodeling through mechanisms mediated by endothelium [113]. In hypertension, hypercholesterolemia, or diabetes, endothelial dysfunction that is considered as a primary manifestation of cardiovascular disease is systemic and is widely attributed to impaired expression of eNOS [114]. Shear stress induces eNOS expression and hence results in greater availability of NO. Since NO is protected vessels from oxidative stress and inflammation, vascular zones with low NO production will be more susceptible to atherosclerosis.

Apolipoprotein E- (ApoE-) deficient mice spontaneously develop atherosclerosis when fed on high cholesterol diet. When a tapered cast was placed around the carotid artery in ApoE-deficient mice, low NO production and advanced lesions were observed in the atherosclerosis-prone flow separation region located immediately downstream of the cast [115]. Further studies on ApoE-deficient mice with implanted cast showed that shear stress type (such as lowered stress or oscillatory stress) is an essential condition for establishing plaque phenotype. Lowered shear stress induces larger lesions with a vulnerable plaque phenotype, whereas vortices with oscillatory shear stress induce stable lesions [116].

Atherosclerosis-resistant and atherosclerosis-susceptible arterial regions were shown to have differentiated endothelial-specific transcriptome patterns, with global expression shifted towards upregulation of proinflammatory and procoagulant genes in susceptible regions and towards upregulation of antioxidant and anticoagulant genes in resistant regions [111, 117]. Disturbed blood flow also contributes to the formation of atherosclerosis-prone vascular regions. Indeed, coordinated regulation of gene expression in ECs in response to local shear stresses should determine regional endothelial phenotypes that protect or predispose to atherosclerosis [118, 119]. Unidirectional and laminar shear stress correlates with transcript profiles considered protective (e.g., antioxidative, anti-inflammatory, and antiproliferative) [120].

Recently, researchers implemented comparative quantification of global miRNA transcriptome in endothelium derived from atheroresistant and atherosusceptible arterial regions in order to identify miRNAs whose expression could distinguish between resistant and susceptible regions. A role of miR-10a in establishing proinflammatory endothelial phenotype in atherosusceptible aortic endothelium was revealed [121]. In swine aorta, site-specific miR-92a regulation of expression of endothelial antiatherogenic transcription Krüppel-like factors- (KLF-) 2 and 4 was shown to contribute to endothelial phenotype heterogeneity associated with regional atherosusceptibility and protection* in vivo* [122].

Upregulation of the miR-221/222 cluster in arterial ECs appears to be proatherogenic since these miRNAs downregulate eNOS and inhibit angiogenesis essential for vascular repair. In addition, miR-221 targets PI3KR1 that is involved in the regulation of PI3K/Akt-mediated signaling that was shown to stimulate the atheroprotective transcription factor Nrf2 (nuclear factor (erythroid-derived 2)-like 2) [120]. Notably, expression of Nrf2 in ECs is induced by mechanical forces (e.g., by shear stress) [123] and contributes to formation of the endothelial regional atherosclerosis-resistant phenotype in vessels [120]. In ECs, Nrf2 drives expression of several antioxidant genes and downregulates several inflammatory mediators such as monocyte chemoattractant protein- (MCP-) 1 and vascular cell adhesion molecule-1 (VCAM-1) therefore enhancing antioxidant and anti-inflammatory properties of the arterial epithelium [124]. Furthermore, expression levels of circulating miR-221/222 are dynamically modulated by blood flow since they were shown to be upregulated by acute exercise before and after sustained training [107]. Indeed, miR-221/222 have a promising potential to be used for mapping atherosusceptible regions in apparently normal arteries.

miR-221 and miR-222 could be upregulated in the vascular epithelium and VSMCs in angiotensin II dependent manner [32, 39]. Pathological activation of the renin-angiotensin system is an established cardiovascular risk factor associated with the development of endothelial dysfunction and essential hypertension [125]. Vascular inflammation, an essential feature of atherosclerosis, also stimulates expression of both miRNAs in the vascular wall [30, 32, 60]. High glucose was shown to induce elevated levels of miR-221 in ECs that impairs normal endothelial function [37]. Furthermore, in blood serum of patients with metabolic syndrome, increased concentrations of circulating miR-221 were detected [126]. Given a prominent role of diabetic hyperglycemia and cardiometabolic stress in the development of cardiovascular pathology, increased expression of the miR-221/222 cluster in vascular tissues should predispose to atherosclerosis and increase risk of endothelial dysfunction and cardiovascular disease. In susceptible individuals, arterial expression of miR-221/222 may be enhanced even in early atherosclerotic stages such as preclinical atherosclerosis [127].

The initial stage of atherosclerosis is characterized by proinflammatory recruitment of leukocytes to activated ECs [128]. Since miR-221 was shown to suppress adhesiveness of ECs through inhibiting several adhesion-related molecules such as VCAM-1, CD47, and PAK1 [59, 129], it seems to initially play an atheroprotective role and is upregulated as a part of the adaptive inflammatory-driven response in order to maintain endothelial homeostasis and quiescent phenotype and prevent activation of arterial ECs. However, continuous proinflammatory stimulation of ECs in early atherosclerosis further upregulates miR-221/222 that in turn promotes switch in the adaptive reaction to the maladaptive proatherogenic response. In atherosclerosis, elevated miR-221/222 inhibits angiogenic activation and therefore limits recruitment and availability of EPCs for vascular repair [70, 130]. In patients with coronary artery disease (CAD), increased serum levels of miR-221 were reported to inversely correlate with numbers of EPCs [131]. Treatment with atorvastatin, a lipid-lowering agent, results in decreasing miR-221 levels and restoring EPC numbers in CAD patients [131]. In diabetes-related atherosclerosis, hyperglycemia and advanced glycation-end products (AGEs) contribute to endothelial dysfunction and cell cycle arrest. AGE-induced downregulation of miR-221/222 in vascular ECs leads to subsequent activation of their targets p27^Kip1^ and p57^Kip2^ that in turn suppress cell cycle progression [132].

Serum miR-221 was reported to be significantly increased in internal mammary arteries of diabetic subjects with coronary artery bypass grafts [133], in patients with carotid atherosclerosis and CAD patients [131]. However, some reports showed reduced levels of serum miR-221 in subjects with atherosclerosis obliterans [134] and atherosclerotic patients with subclinical hypothyroidism [135]. In advanced atherosclerosis, long-term chronic inflammation, which occurs parallel with remodeling of the intimal extracellular matrix [136], was found to downregulate expression of the miR-221/222 cluster in vascular ECs [60]. Probably, suppression of miR-221/222 could precede the neointima formation in atherosclerotic vessels when the recruitment of ECs is needed to support neoangiogenesis. Inflammatory factors suppressing miR-221/222 in ECs should be detected. Interferon-*γ* (IFN-*γ*), a proinflammatory cytokine, inhibits miR-221 expression in cholangiocytes but it is unknown whether IFN-*γ* is involved in miR-221 suppression in ECs [137]. Another inflammatory cytokine that is likely to regulate vascular expression of miR-221 in atherosclerosis is high-mobility group B1 (HMGB1; alarmin) [138]. HMGB1 was found to play a remarkable role in atherosclerosis-associated inflammation by signaling via receptor for advanced glycation end products (RAGE) and Toll-like receptors [139].

### 4.2. miR-221/222 Cluster Induces VSMC-Mediated Neointima Formation and Contributes to Vascular Calcification in Atherosclerosis

Like PDGF, angiotensin II is able to induce miR-221/222 [39], followed by VSMC proliferation and hypertrophy [140]. Angiotensin II induced activation of miR-221/222 expression in VSMCs is more attributable to vascular pathology such as hypertension, restenosis, or atherosclerosis and is accompanied with induction of the proinflammatory microenvironment [141]. In VCMCs, angiotensin II stimulates expression of miR-221 through binding to angiotensin II type 1 receptor (AT1R) that mediates activation of cAMP response element-binding protein (CREB), a transcription factor [36]. miR-221 was found to show a positive feedback on its own expression by suppressing RASA1 (RAS p21 protein activator 1), a CREB inhibitor. miR-221 also negatively regulates PTEN (phosphatase and tensin homolog), a suppressor of Akt/PI3K signaling that induces NF-*κ*B-mediated production of inflammatory chemokine MCP-1 [36]. Indeed, angiotensin II dependent downregulation of PTEN results in increased expression of proinflammatory mediators and activates proliferation, hypertrophy, and migration of VSMCs [142].

PDGF production and PDGF-dependent signaling were shown to be activated in vascular proliferative diseases including atherosclerosis and restenosis and therefore could drive VSMC recruitment in vascular remodeling [143]. Indeed, PDGF-induced miR-221/222 upregulation followed by suppression of the cell cycle regulator p27^Kip1^ in VSMCs could contribute to atherogenesis because loss of p27^Kip1^ was reported to aggravate atherosclerosis in ApoE-deficient mice [144, 145]. In ApoE-deficient mice, expression of transgenic ApoE, an essential component of serum high density lipoproteins (HDL) and triglyceride-enriched lipoproteins that possess atheroprotective properties, negatively regulates miR-221/222 and restores p27^Kip1^ expression in VSMCs thereby preventing pathological recruitment of these cells in atherosclerosis-related vascular remodeling. ApoE was found to downregulate miR-221 and miR-222 in a Cox-2 (prostaglandin-endoperoxide synthase)/prostacyclin/inositol monophosphate-dependent manner [146]. ApoE could also prevent dedifferentiation of VSMCs by suppressing PDGF-mediated entry to S-phase through activation of inducible NOS [147] or interaction with perlecan, an extracellular matrix protein [148]. However, these mechanisms are dependent on specific ApoE isoforms while all three isoforms of ApoE are able to suppress miR-221/222 through Cox-2-dependent pathway [146]. Indeed, due to capacity of all ApoE isoforms to regulate miR-221/222-dependent input to control of VSMC phenotypic plasticity, this mechanism could represent the major way by which ApoE may limit proatherogenic entrance of VSMCs to arterial remodeling.

In the family of CDK protein inhibitors (p15^Ink4A^, p16^Ink4B^, p18^Ink4C^, p19^Ink4D^, p21^Waf1/Cip1^, p27^Kip1^, and p57^Kip2^), three cell cycle regulators (p21^Waf1/Cip1^, p27^Kip1^, and p57^Kip2^) play a central role in control of VSMC differentiation/dedifferentiation because p57^Kip2^ regulates G1/S transition of cell cycle while p21^Waf1/Cip1^ and p27^Kip1^utilize different molecular mechanisms of CDK inhibition in G1-phase [149, 150]. Since miR-221 and miR-222 target two of them (p27^Kip1^ and p57^Kip2^), and this miRNA cluster may have a profound impact in the regulation of the role of VSMCs in proatherogenic neointimal hyperplasia [24, 127].

Furthermore, results recently presented by Mackenzie et al. [34, 151] about supportive effects of miR-221/222 on vascular calcification expand proatherogenic properties of this miRNA cluster. Calcification commonly affects the arterial tree in a variety of diseases and pathological conditions [152–155], with calcification process often being associated with VSMCs [152, 156]. In calcifying VSMCs transfected with miR-221/222 mimics, a significant increase in intracellular calcium accumulation associated with quantitative changes in expression of ectonucleotide phosphodiesterase 1 (Enpp1) and Pit-1 (sodium-dependent phosphate cotransporter) expression was observed [34]. Both proteins are essential for regulation of intracellular phosphate levels and hence are primarily involved in control of calcium phosphate deposits in osteogenesis and pathogenic ectopic mineralization including atherosclerosis-related arterial calcification [157]. Interestingly, when VSMCs were transfected with miR-221 and miR-222 mimics, an increase in calcium deposition was observed in combined treatment but not in individual miR treatments suggesting for synergistic effects of miR-221/222 on vascular calcification [151]. Both miRNAs were shown to contribute to VSMC calcification independently of osteogenic transcription factors Runx2 (runt-related transcription factor 2) and Msx2 (MSH homeobox 2) [34].

Calcification of VSMCs is associated with significant decrease in miR-221/222 levels [151]. Indeed, downregulation of miR-221/222 could induce osteogenic/chondrogenic changes in the VSMC phenotype. These observations are in agreement with the data presented by Li et al. [158] who detected reduced levels of miR-221/222 in sclerotic intima samples from patients with atherosclerosis obliterans compared to the normal vascular tissues. In advanced atherosclerosis, long-term inflammation decreases miR-221/222 in ECs [60]. It is likely that chronic vascular inflammation could also diminish miR-221/222 expression in VSMCs and therefore promote induction of phenotypic changes towards calcifying cells. However, molecular mechanisms by which the miR-221/222 cluster contributes to vascular calcification are widely unknown and indeed should be precisely evaluated in future studies.

### 4.3. Challenges for Analysis of the Roles of miR-221/222 in Functioning of Cells Types That Represent Minor Cell Populations in the Vascular Wall

Current appreciation of a remarkable role of miRNAs in vascular biology of VSMCs and ECs warrants investigation of possible contribution of miR-221/222 to functioning of other cell types residing in the vascular wall, in both homeostatic and pathological conditions. In particular, such a demand is relevant to investigation of vascular dendritic cells representing the most crucial cell type that regulates immune processes in atherogenesis and other vascular pathologies in which immune inflammation plays an important role [159–162]. Accumulating evidence indicates that miRNAs, including miR-221/222, are importantly involved in functioning of dendritic cells in other organs and other pathologies [163–165]. In particular, differentially expressed microRNAs have been reported to regulate plasmacytoid versus conventional dendritic cell development [164]. As vascular dendritic cells (VDCs) represent a minor cell population in the arterial wall [159], obviously there would be some methodological difficulties during the investigation. However, the recent achievements in the visualization of miRNA localization in cells within biological tissue sections by a combination of* in situ* hybridization with immunohistochemistry [166–168] along with the achievements in the development of protocols for the isolation of arterial cells by means of the use of laser capture-microdissection technique [169–171] would assist in investigation of the impact of miR-221/222 in controlling of behavior patterns of dendritic cells in various vascular pathologies.

## 5. Extravasal Functional Effects of miR-221/222 Increasing Cardiovascular Risk

In addition to the direct regulation of function and development of vascular cells, miR-221 and miR-222 exhibit their effects on nonvascular tissues. Some of these functional activities could be related to the development of atherosclerosis or cardiometabolic risk factors that promote atherogenesis. In the adipose tissue, miR-221 was recently found to control fat metabolism by affecting PPAR- (peroxisome proliferator-activated receptor-) dependent pathways and by directly targeting adiponectin receptor AdipoR1 and Ets1 [172]. miR-221 and RNA-binding protein polypyrimidine tract-binding protein (PTB) cooperate in negative posttranslational control of AdipoR1 in muscle and liver of genetic and dietary murine models of obesity that suggests a supportive role of miR-221 in insulin resistance in peripheral tissues [173].

AdipoR1 mediates biological effects of adiponectin, an adipocyte-specific cytokine, mainly focused on the regulation of glucose metabolism (increased glucose uptake, reduced gluconeogenesis) and fat metabolism (activation of lipid catabolism, oxidation of fatty acids, and utilization of triglycerides) [174]. Interestingly, in vascular ECs, AdipoR1 mediates vasculoprotective properties of adiponectin associated with anti-inflammation (inhibition of tumor necrosis factor-*α*- (TNF-*α*-) induced expression of ICAM-1 and NF-*κ*B) and prevention of vascular dysfunction [175]. However, it is unclear whether miR-221 targets AdipoR1 in the vascular endothelium. In cultured preadipocytes, leptin and proinflammatory cytokine TNF-*α* suppress expression of miR-221 [172]. Similarly, Chou et al. [176] showed decreased levels of miR-221 in MSCs derived from the adipose tissue of obese women, and miR-221 expression was negatively correlated with levels of TNF-*α* mRNA in obese adipocytes. Indeed, TNF-*α* could downregulate miR-221 in adipocytes.

Overall, miR-221, which is upregulated in obese individuals [172], is involved in insulin resistance and metabolic syndrome, for example, two metabolic conditions that greatly increase atherosclerotic risk. In morbidly obese individuals, blood levels of miR221/222 are markedly increased [177]. In adipocytes, miR-221 contributes to the regulation of physiological network involved in fatty acid metabolism by targeting several proteins including fatty acid synthase (FASN), an enzyme overexpressed in the adipose tissue in obesity and type 2 diabetes [178]. In obese people, adipocytes have activated fat metabolism associated with lipolysis and enhanced release of free fatty acids to blood. Fatty acids promote insulin resistance via suppressing insulin signaling by stimulating serine protein kinases that phosphorylate insulin receptor substrates (IRS) and disrupt the downstream transduction of signal from the insulin receptor [179].

Serum miR-221 levels were shown to be elevated in women affected with metabolic syndrome although no significant correlations were observed between blood miR-221 concentrations and cardiometabolic risk factors [126]. Diabetic hyperglycemia and products of advanced nonenzymatic oxidative glycation that are significantly increased in diabetic blood were shown to stimulate expression of miR-221/222 in the vascular wall [37, 133, 180]. Indeed, upregulated expression of the miR-221/222 cluster in vessels of people with obesity, metabolic syndrome, insulin resistance, hypertension, and type 2 diabetes increases cardiovascular risk and promotes the development of atherosclerosis through endothelial dysfunction and neointimal hyperplasia. The proatherogenic role of miR-221/222 is supported by the fact of suppressing effects of lipid-lowering agents (atorvastatin), antidiabetic drugs (metformin), and garlic preparations on vascular expression of this miRNA cluster [58, 130, 131, 133].

## 6. Clinical Potential of miR-221/222

Serum levels of circulating miR-221 were shown to be significantly changed in several vascular and metabolic pathologies including hypertension, obesity, metabolic syndrome, CAD, and carotid atherosclerosis that make this miRNA a potential diagnostic biomarker. In patients with acute coronary syndrome, miR-221 was found to be significantly increased only in platelets and PMBCs of those with non-ST-segment elevation (NSTEMI) myocardial infarction but not with a ST-segment-elevation myocardial infarction (STEMI) suggesting a potential diagnostic value of this miRNA for distinguishing between STEMI and NSTEMI [181]. A phenomenon of the blood flow-mediated regulation of expression of miR-221 in the vascular wall should be studied in detail because differential expression of miR-221 in arterial wall harbors a promise of identification of atheroprone and atheroresistant vascular regions in asymptomatic patients or subjects with subclinical atherosclerosis that is important for early diagnosis and prophylaxis of cardiovascular disease.

To date, the major progress on the way of clinical utility of miR-221/222 in cardiovascular medicine was achieved in the field of stem cell therapy for cardiovascular diseases. The miR-221/222 cluster is critically involved in the regulation of myogenesis and function of myocardium [173, 182–184]. Both in myoblasts and myotubules, miR221/222 expression was shown to be under control of the Ras-MAPK pathway and inversely correlated with levels of p27^Kip1^, a common target for these miRNAs [182]. The cluster could also contribute to several heart pathologies including myotonic dystrophy type 2 [185] and hypertrophic cardiomyopathy (through targeting p27^Kip1^, a cardiac hypertrophic suppressor in cardiomyocytes) [35, 59, 186].

Treatment with a prosurvival cocktail consisting of lentivirus constructs carrying the precursor of miR-21, miR-24, and miR-221 significantly improved survival and engraftment of cardiomyocyte precursors after transplantation to the mouse cardiac muscle [187]. In part, the prosurvival effect of the miRNA cocktail was supported by inhibition of BIM/BCL2L11 (BCL2-like 11), a critical apoptotic activator, which is a common target for all three miRNAs [187]. In advanced oxidative stress, miR-221 was demonstrated to cooperate with superoxide dismutase-2 in unacylated ghrelin-driven muscle repair after oxidative injury by downregulation of p57^Kip1^ that in turn promotes cell cycle progression [180]. In addition, miR-221 was found to support survival of cocultures of primary rat neonatal ventricle cardiomyocytes and MSCs transduced with GATA-4 (a critical transcription factor for proper mammalian cardiac development) in hypoxic conditions. The miR-221-mediated cardioprotection was achieved via downregulation of p53 upregulated modulator of apoptosis (PUMA). Interestingly, compared to cardiomyocytes, cultured MSCs developed significantly higher expression levels of miR-221 and supported cardiomyocyte viability by shedding miR-221-containing microvesicles [188]. Thus, miR-221 show marked cardioprotective and antiapoptotic properties that would be beneficial for increasing efficiency of cardiac cell transplantation and heart regeneration.

## 7. Conclusions

Likewise other miRNAs [189–194], the miR-221/222 cluster plays a remarkable role in vascular biology and vascular pathology. In the vascular tissue, these miRNAs exhibit cell-specific effects by supporting dedifferentiation proliferation and migration of VSMCs while inhibiting proliferation and motility of ECs. In different muscle lineage cells, miR-221 and miR-222 show similar effects by activating regenerative properties through targeting key cell cycle regulators p21^Waf1/Cip1^, p27^Kip1^, and p57^Kip2^ involved in induction of expression of contractile proteins. In contrast to antiangiogenic activity in vascular ECs, miR-221/222 were shown to support tumor-associated neoangiogenesis and invasion in many cancers including hepatocellular carcinoma [195], breast cancer [196], lung cancer [197], colorectal cancer [198], and other epithelial cancers. Tumorigenic function of miR-221/222-mediated is mainly attributed to targeting several key tumor suppressors such as p21^Waf1/Cip1^, p27^Kip1^, p57^Kip2^, PTEN, and PUMA [199, 200].

In epithelial cancers, the miR-221/222 cluster is involved in induction of EMT, a mechanism that leads to phenotypic changes in cancer cells and cancer-associated ECs towards increased dedifferentiation, mobility, adhesion, and invasiveness [201]. It seems that tumor progression to metastasis stage does not happen occasionally and chaotically but is induced in a coordinated manner, with burst in tumor neovascularization. EMT was shown to play a central role in vasculogenic mimicry, a new pattern of tumor microcirculation, in which cancer cells acquire properties of vascular cells having tumor and endothelial phenotypes with maintenance of stem cell-like characteristics to form capillary-like structures in the tumor mass [202]. Hypoxia promotes vasculogenic mimicry formation by inducing EMT [203]. Indeed, activation of the proinvasive EMT mechanism should be involved in the control of tumor-associated neovascularization since tumor microvascular network facilitates mobilization of tumor cells to circulation and further expansion [204]. For example, miR-106b/93 and miR-221/222 are upregulated in gastric cancer tissues and control suppression of CDK protein inhibitors in a coordinated manner thereby stimulating tumor invasion and progression [199]. EMT is absent in normal and diseased noncancer vascular tissues and therefore does not influence neoangiogenesis. Therefore, upregulated production of miR-221/222 in terminally differentiated vascular ECs will inhibit angiogenic activation whereas downregulation of this miRNA cluster will favor induction of neoangiogenesis.
